# Effects of Cellulose Nanofiber Reinforcement on the Properties of Three-Dimensional-Printed Denture Base Resin: An In Vitro Study

**DOI:** 10.3390/ma19132891

**Published:** 2026-07-06

**Authors:** Xiangyu Ren, Tamaki Hada, Keyu Qi, Masanao Inokoshi, Motohiro Uo, Manabu Kanazawa

**Affiliations:** 1 Gerodontology and Oral Rehabilitation, Graduate School of Medical and Dental Sciences, Institute of Science Tokyo, Tokyo 152-8550, Japan; x.ren.gerd@tmd.ac.jp (X.R.); kikayoku@outlook.com (K.Q.); m.kanazawa.gerd@tmd.ac.jp (M.K.); 2 Oral Devices and Materials, Graduate School of Medical and Dental Sciences, Institute of Science Tokyo, Tokyo 152-8550, Japan; m.inokoshi.gerd@tmd.ac.jp; 3 Oral Science Center, Institute of Biomedical Engineering, Institute of Science Tokyo, Tokyo 152-8550, Japan; 4 Advanced Biomaterials, Graduate School of Medical and Dental Sciences, Institute of Science Tokyo, Tokyo 152-8550, Japan; uo.abm@tmd.ac.jp; 5 Clinic of General, Special Care and Geriatric Dentistry, Center for Dental Medicine, University of Zurich, 8006 Zurich, Switzerland

**Keywords:** cellulose nanofibers, three-dimensional-printed denture base resin, digital light processing, mechanical properties, color stability

## Abstract

**Highlights:**

- Low-dose CNF (1.0 wt%) significantly improved the flexural strength and surface hardness of 3D-printed denture base resin while maintaining favorable color stability.
- Higher CNF concentrations increased water sorption and promoted agglomeration or void formation, suggesting the importance of optimizing filler content.
- CNF is a promising bio-based reinforcing filler for enhancing the durability and sustainability of digitally fabricated denture base materials.

**Abstract:**

Although the use of three-dimensional-printed (3D-printed) denture base resins is becoming increasingly widespread in digital dentistry, information regarding the reinforcing effects of cellulose nanofibers and their most favorable concentration remains limited. We evaluated the effects of adding cellulose nanofibers (CNFs, 0–2.0 wt%) on the mechanical and physical properties of three-dimensional-printed denture base resins. The addition of cellulose nanofibers improved flexural strength (FS) and Vickers hardness (HV), with more balanced performance observed at low concentrations; no significant differences were observed in flexural modulus. Color stability did not show a linear concentration-dependent trend, and the 1.0 wt% group exhibited the most favorable overall performance. Water sorption slightly increased at higher CNF concentrations. SEM observations revealed relatively uniform CNF dispersion at lower concentrations, whereas agglomeration and void formation occurred at higher concentrations. Appropriate CNF incorporation may be a promising strategy for reinforcing 3D-printed denture base materials.

## 1. Introduction

Denture base prostheses are widely used to improve oral function, esthetics, and quality of life for patients with partial or complete edentulism [1]. Despite advancements in dental materials for denture bases, fractures, surface degradation, and poor dimensional stability are common and may affect the longevity of restorations and increase maintenance requirements [2,3]. Therefore, enhancing the durability of denture base materials remains a key objective in prosthodontic research [4].

The application of additive manufacturing or three-dimensional (3D) printing technologies in prosthodontics has recently advanced denture fabrication workflows, improving efficiency, reproducibility, and material utilization [5,6,7]. Among the available technologies, digital light processing (DLP) allows the fabrication of high-resolution restorations and materials with smooth surfaces, making it particularly suitable for denture base production [8,9]. Despite these advantages, denture base materials produced through digital workflows have shown lower fracture resistance and flexural performance than conventional heat-polymerized denture base resins [10,11,12,13]. Additionally, 3D-printed dentures are more susceptible to staining and discoloration from colored foods and beverages, such as curry and coffee, leading to reduced long-term color stability and compromised patient satisfaction [14,15,16,17]. Such limitations may compromise the long-term durability of digitally fabricated dentures in clinical use [18]. Therefore, improving the mechanical performance of 3D-printed denture base resins remains an important objective for expanding their long-term clinical applicability.

Filler reinforcement using materials such as conventional fibers and nanoparticles has been increasingly explored as a method to enhance the structural strength of polymer-based dental materials [19,20,21]. Recent studies have proposed enhancing the mechanical performance and durability of denture base resins by incorporating nanofillers [22,23,24,25,26]. Various inorganic and carbon-based nanofillers, including silica nanoparticles, zirconia, and carbon fibers, have demonstrated reinforcement potential, improving flexural properties and wear resistance [27]. However, these materials usually cause aggregation, uneven dispersion, and compromised esthetics owing to reduced translucency or uneven optical properties [28,29]. Moreover, certain carbon-based fillers may pose risks such as biocompatibility concerns, processing difficulties, and long-term biological safety issues [30]. These limitations emphasize the need for new types of reinforcing fillers that can enhance mechanical properties while maintaining esthetic appeal and biocompatibility.

Cellulose nanofibers (CNFs) have been investigated as naturally sourced nanofillers because of their mechanical properties, aspect ratio, biocompatibility, and renewability [31,32,33,34]. Their networking ability may provide greater reinforcing potential than cellulose nanocrystals (CNCs) [35]. Compared with other nanomaterials, CNFs are considered to have a more favorable safety profile, supporting their potential use in dental materials. Their abundant hydroxyl groups enable strong interfacial interactions with polymer matrices, facilitating effective stress transfer even at low filler concentrations [36,37]. In addition, CNFs can be incorporated into resin matrices through a simple mixing process without requiring specialized equipment or surface treatment, which may enhance their practicality. Unlike some synthetic nanomaterials and microplastics that are associated with environmental concerns and exhibit relatively high densities, CNFs are derived from renewable resources and are lightweight, making them a potentially advantageous material from both clinical and environmental perspectives. Based on these properties, CNFs have potential for developing denture base materials reinforced with renewable bio-based nanofillers [38,39,40].

CNF incorporation enhances the mechanical properties of various polymers, including flowable dental composite resins, medical-grade light-curing polymers, and polymethyl methacrylate denture base resins [41,42,43,44]. However, because of fundamental differences in viscosity, polymerization mechanics, and filler dispersion behavior between thermosetting systems and light-curing polymers, the reinforcement effects of these materials cannot be directly extrapolated to 3D-printed denture resins [45]. Although previous studies have explored the reinforcing potential of CNFs in dental and polymer materials, few have comprehensively evaluated the concentration-dependent effects of CNF on the mechanical properties and physicochemical behavior of resins used in additive manufacturing for denture bases.

In this in vitro study, we aimed to examine denture base materials incorporating cellulose nanofibers and fabricated via additive manufacturing and to identify the concentration range (0–2.0 wt%) that provides more balanced material performance. The null hypothesis was that CNF addition does not significantly affect the flexural strength, flexural modulus (FM), surface hardness, color stability, water sorption, or solubility of 3D-printed denture base resins at different concentrations.

## 2. Materials and Methods

### 2.1. Materials and Experimental Design

**Figure 1:**
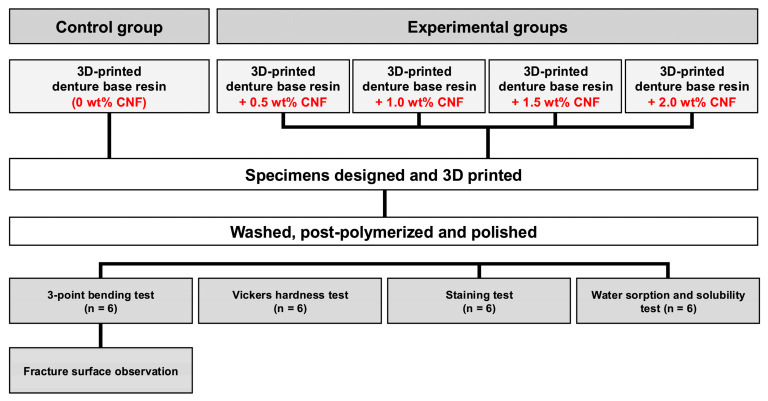
Schematic of experimental design. CNF, cellulose nanofiber.

Figure 1 summarizes the overall experimental workflow, including specimen fabrication, mechanical testing, physical property evaluation, and microstructural characterization. Information regarding the materials included in the investigation is provided in Table 1.

Sample size estimation was performed using G*Power, version 3.1.9.7 (Heinrich-Heine-Universität Düsseldorf, Düsseldorf, Germany). The sample size was initially calculated based on the effect size reported in a previous study [46]. Using the FS and standard deviation (SD) values from that study, the effect size was estimated to be 2.19. With an α-level of 0.05 and a 1-β (power) level of 0.95; the minimum required sample size was calculated to be six specimens per group. In addition, our pilot study showed FS values of 62.8 ± 9.5 MPa in the control group and 87.5 ± 13.2 MPa in the 2.0 wt% CNF group, yielding an estimated effect size of 2.14. Based on this effect size, power analysis also indicated that six specimens per group would be sufficient, thereby confirming the sample size determined from the previous study. A 3D-printed denture base resin (dima Print; Kulzer GmbH, Hanau, Germany) containing approximately 30–50% urethane dimethacrylate, 3–10% propylidynetrimethyl trimethacrylate, 3% diphenyl(2,4,6-trimethylbenzoyl) phosphine oxide, and 1% mequinol was used in this study, as specified in the manufacturer’s Safety Data Sheet (SDS). The CNFs (WFo-UNDP; Sugino Machine, Toyama, Japan) had a diameter of approximately 7–12 nm. Based on the manufacturer’s SDS, the CNF dry powder was produced via mechanical fibrillation technology from biomass fibers of cellulose, chitin, chitosan, and silk.

### 2.2. Preparation of Nanocomposites and Fabrication of Specimens

The samples were designed using computer-aided design software (Geomagic Freeform, version 2022 1.32; Hexagon, Stockholm, Sweden) and were placed horizontally on the build platform. Next, the design data were exported as a standard tessellation language file and transferred to a 3D printer prior to printing. Table 2 shows the 3D printing and post-processing parameters.

In this study, unmodified additive manufacturing resin was used as the control group (0 wt%), while cellulose nanofibers were incorporated at concentrations of 0.5, 1.0, 1.5, and 2.0 wt%. The selected concentration range was based on previous studies on CNF-reinforced 3D polymer materials [41]. To systematically evaluate concentration-dependent effects while maintaining a practical experimental design, the intermediate concentrations were set in increments of 0.5 wt%. The selected CNF concentration was determined based on previous studies and preliminary considerations regarding dispersion stability and printability. Higher CNF loading may lead to increased resin viscosity and particle aggregation, which could negatively influence fabrication accuracy and overall material performance [19]. No chemical or surface modification treatment was performed on the CNFs prior to incorporation into the resin matrix. The 3D-printed resin-CNF mixture was dispersed in a homogenizer (HG-200; Hsiangtai Machinery Industry, New Taipei City, Taiwan) for 5 min at 100 rpm and positioned in a vacuum chamber connected to a diaphragm dry vacuum pump (DA-40S; ULVAC, Chigasaki, Kanagawa, Japan) to facilitate the elimination of air bubbles. Subsequently, the mixture was transferred to the tank of the 3D printer (cara Print 4.0; Kulzer) using DLP technology to fabricate the specimens with a layer thickness of 100 μm. This 3D printer, which delivers a 405 nm wavelength during printing, was selected because of its high resolution, widespread use in the field of dental additive manufacturing, and compatibility with the resin system used in this study. After 3D printing, the samples were washed with a 99% isopropyl alcohol solution for 10 min and post-polymerized at 60 °C for 20 min using a post-polymerizing device (Hilite Power 3D; Kulzer). Definitive finishing was completed with a grinder (DialapML150P; Maruto, Tokyo, Japan) using #800, #1000, and #1200 waterproof abrasive papers and an alumina-based abrasive to standardize each specimen’s dimensions.

### 2.3. Three-Point Bending Test

**Figure 2:**
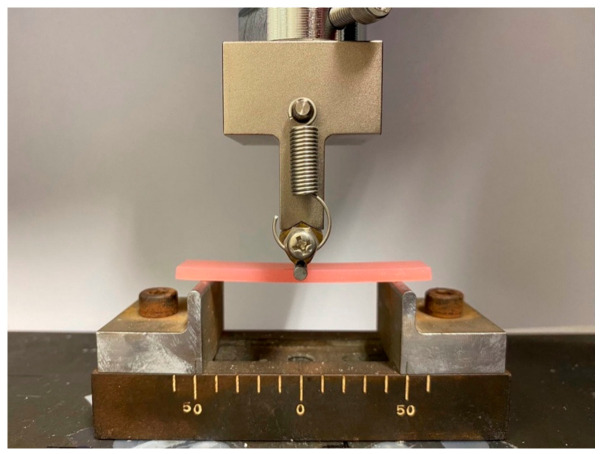
Setup of the three-point bending test.

After additive manufacturing, the specimens were immersed in 99% isopropyl alcohol for 10 min and then post-cured for 20 min at 60 °C using a post-curing device (Hilite Power 3D; Kulzer). Their flexural properties were evaluated using the three-point bending method in accordance with ISO 20795-1:2013 [47]. Prior to testing, all specimens were soaked in ultrapure water at 37 °C for 50 h. Rectangular specimens measuring 64 × 10 × 3.3 mm (*n* = 6) were loaded using a universal testing machine (AG-Xplus; Shimadzu, Kyoto, Japan) with a 50 mm gauge length and crosshead speed of 5 mm/min. A loading force was applied via a ram until the specimen fractured (Figure 2). FS (MPa) and FM (GPa) were determined according to the following formulas:

$$FS\;=\;3Fl/2bh^{2}$$

$$FM=Fl^{3}/4bh^{3}d$$

where *F* represents the applied load (N) within the linear portion of the load–deflection curve, *l* indicates the support span (mm), *b* and *h* correspond to specimen width and thickness (mm), respectively, and d denotes deflection (mm).

### 2.4. Fractured Surfaces Microscopy

Following the three-point bending procedure, fractured surfaces were subjected to fractographic observation with scanning electron microscopy (SEM; SM-7900F; JEOL, Tokyo, Japan). Prior to imaging, the specimens received a platinum–palladium coating. Micrographs were acquired at ×500 and ×2000 magnifications with 5 kV accelerating voltage and 10 mm working distance.

### 2.5. Vickers Hardness Test

Vickers hardness tests were conducted on disc-shaped specimens (*n* = 6) with a diameter of 10 mm and a thickness of 2.0 mm using a Vickers hardness testing machine (MVK-H2; Akashi, Tokyo, Japan). A load of 300 gf (0.3 kgf) was applied for 15 s, and the hardness values were reported as HV_0.3_. Five indentations were made on each specimen, and the mean value was calculated based on a previously described method [48]. The Vickers hardness value (HV) was calculated as follows:

$$VHN=1.854\cdot F/D^{2}$$

where *F* refers to the applied load in newtons (N; converted from kgf using 1 kgf = 9.807 N), and *D* corresponds to the distance between opposing vertices of the tetragonal indentation (mm).

### 2.6. Staining Test

The staining medium was prepared by dissolving 4 g of curry powder (S&B Shokuhin, Tokyo, Japan) in 350 mL of warm ultrapure water. Disc-shaped samples measuring 20 mm in diameter and 2.5 mm in thickness (*n* = 6) were then immersed in the solution at 37 °C for 7 days, and the staining medium was refreshed every 24 h. Subsequently, the samples’ color before immersion was compared with that after 3 days and after 7 days of immersion in the curry solution. The specimens’ colors were measured on a white background (L* = 92, a* = 0.15, and b* = 1.6) using a colorimeter (CR-13; Konica Minolta, Tokyo, Japan). Color measurements were performed at three locations per specimen, and the mean values (L*, a*, and b*, as defined by the CIE color system) were used as the representative color values. The color stability (ΔE*) was calculated as follows:

$$\Delta E^{*}\;=\;[{\left(\Delta L^{*}\right)}^{2}\;+\;{\left(\Delta a^{*}\right)}^{2}\;+\;{\left(\Delta b^{*}\right)}^{2}{]}^{1/2}$$

### 2.7. Water Sorption and Solubility Tests

Water sorption (W_sp_) and water solubility (W_sl_) tests were conducted following the ISO 20795-1:2013 standard [24,26] using disc-shaped specimens with a diameter of 50 mm and a thickness of 0.5 mm (*n* = 6). A precision analytical balance (TVN480DA; Advantec, Irvine, CA, USA) with an accuracy of 0.1 mg was used to record three mass values. The specimens were initially stored in a desiccator containing dried silica gel at 37 °C for 24 h and subsequently transferred to fresh silica gel at 23 °C for 60 min. Once the mass variation remained within 0.2 mg, the value was defined as *m*_1_. The samples were then immersed in ultrapure water at 37 °C for 7 days. After removal from the water, surface moisture was eliminated by wiping and gentle air shaking, and the resulting mass was recorded as *m*_2_. Reconditioning was subsequently performed in a desiccator at 37 °C until a stable mass was obtained for each specimen. This was recorded as the definitive mass (*m*_3_). The W_sp_ (μg/mm^3^) and W_sl_ (μg/mm^3^) were calculated using the following formulas, where *V* represents the volume of each specimen (mm^3^):

$$W_{sp}\;=\;(m_{2}\;-\;m_{3})/V$$

$$W_{sl}=(m_{1}-m_{3})/V$$

### 2.8. Statistical Analysis

Statistical analysis was conducted using IBM SPSS Statistics version 30.0 (IBM, Armonk, NY, USA), with the significance level set at α = 0.05. Normality of distribution and homogeneity of variance were examined using the Shapiro–Wilk test and Levene’s test, respectively. Tukey’s multiple comparison test was used to compare the mean values of FS, W_sp_, and W_sl_, whereas Welch’s analysis of variance was used for FM. A linear mixed-effects model was used to compare the mean values of ΔE*, followed by post hoc analysis with Bonferroni correction.

## 3. Results

### 3.1. Three-Point Bending Test

Table 3 shows the mean and SD of FS for the control (0 wt% CNF) and various CNF concentration groups. The control group exhibited the lowest FS value (*p* < 0.001). FS increased markedly at 0.5 wt% CNF but showed no significant difference across the 1.0, 1.5, and 2.0 wt% CNF groups (*p* > 0.05). The highest FM value was observed in the 1.5 wt% CNF group (3.2 GPa); however, Welch’s analysis of variance demonstrated no statistically significant intergroup differences (*p* > 0.05).

### 3.2. Fracture Surface Analysis

**Figure 3:**
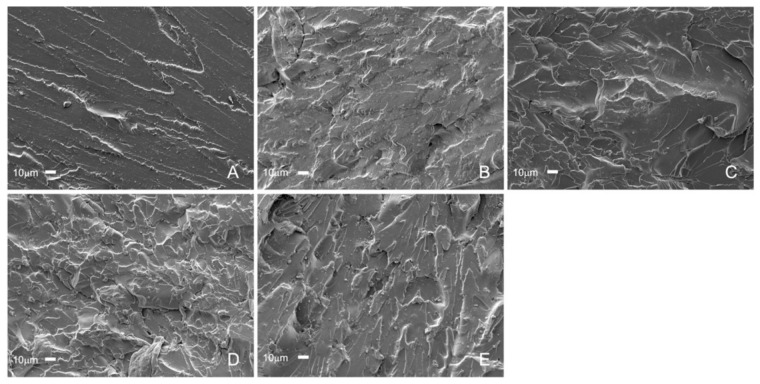
Scanning electron microscopy images show different fractography characteristics of different cellulose nanofiber concentrations in the three-dimensional printed denture base resin (magnification ×500). (**A**) 0 wt% (control), (**B**) 0.5 wt%, (**C**) 1.0 wt%, (**D**) 1.5 wt%, and (**E**) 2.0 wt%.

**Figure 4:**
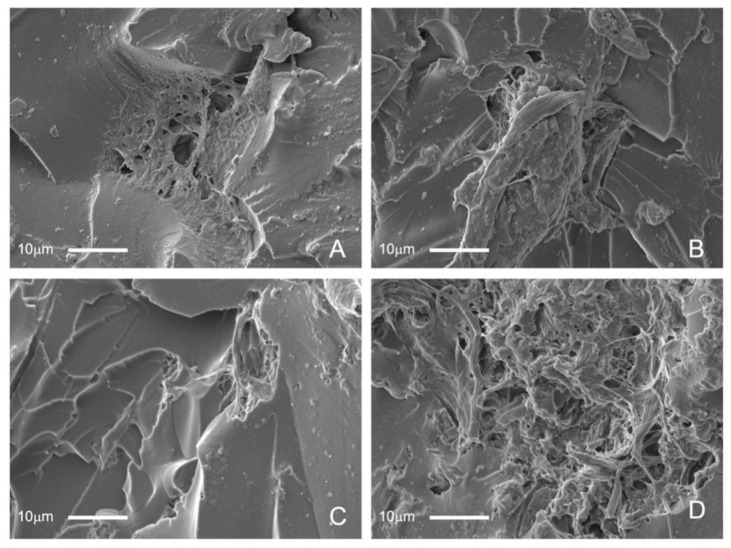
Scanning electron microscopy images show a web-like structure of different cellulose nanofiber concentrations in the three-dimensional printed denture base resin (magnification ×2000). (**A**) 0.5 wt%, (**B**) 1.0 wt%, (**C**) 1.5 wt%, and (**D**) 2.0 wt%.

SEM observation revealed differences in the fracture surface morphology among the groups (Figure 3). The control group exhibited a relatively flat and smooth fracture surface (Figure 3A). In contrast, CNF-containing specimens (Figure 3B–E) displayed rougher fracture surfaces with lamellar and web-like structures that were absent in the control group. In specimens with higher CNF concentrations, irregular surface features and pore-like structures were observed (Figure 4), suggesting a less homogeneous microstructure.

### 3.3. Vickers Hardness Test

Table 4 shows the mean and SD of HV_0.3_ for the control and various CNF concentration groups. The 1.0 (22.5) and 1.5 wt% (22.2) CNF groups exhibited significantly higher HV_0.3_ values (*p* < 0.001). This indicated a marked increase in the HV_0.3_ values compared with those in the control, 0.5 wt%, and 2.0 wt% groups (*p* < 0.001), without any significant differences among these groups (*p* > 0.05).

### 3.4. Staining Test

A linear mixed model ΔE* after curry staining was analyzed with CNF concentration and staining days as fixed effects. The results indicated significant effects among both the groups and immersion durations (*p* < 0.001). ΔE* values varied with increasing CNF concentrations and staining time, and a significant interaction was found between the two factors (*p* = 0.016), indicating that the effect of CNF concentration on color change depends on the staining duration (Table 5).

**Table 5:** Linear mixed model results for color stability (ΔE*) values.

Source	df Numerator	df Denominator	F Value	*p*-Value
Intercept	1	39.687	8304.468	<0.001
CNF concentration	4	39.687	27.397	<0.001
Days	1	39.687	76.082	<0.001
CNF concentration × days	4	39.687	3.471	0.016

df, degrees of freedom; CNF, cellulose nanofiber. Pairwise comparisons revealed that on day 3, ΔE* values were lower in all the CNF groups than in the control group, with the 1.0 wt% group showing the greatest difference. All experimental groups showed increased ΔE* values after 7 days of immersion, with significant differences identified relative to the control group (*p* < 0.001) (Figure 5 and Figure 6). The 1.0 wt% CNF group showed the lowest value (21.3).

**Figure 5:**
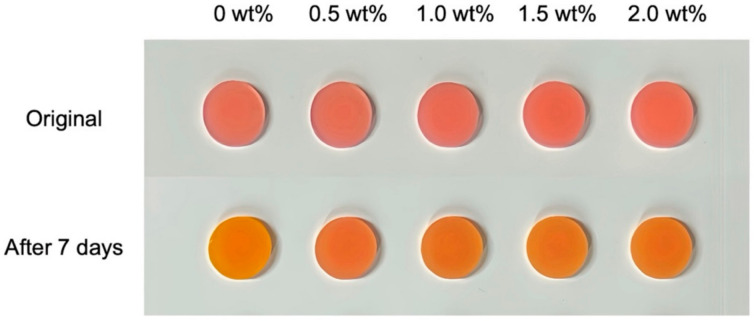
Representative photographs of cellulose nanofiber (CNF)-reinforced three-dimensional (3D)-printed denture base resin specimens before and after 7 days of immersion in curry solution. The images provide a visual comparison of discoloration among the experimental groups and complement the color stability (ΔE) results presented in Figure 6.

**Figure 6:**
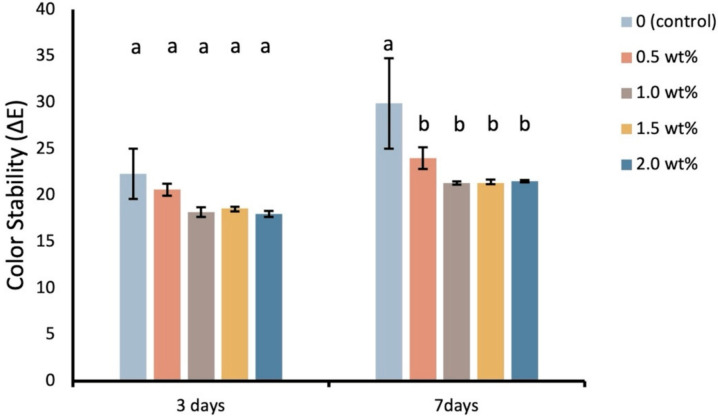
Graph summarizing the linear mixed-effects model results for color change in cellulose nanofiber-reinforced three-dimensional printed resin after staining in curry solution (*p* < 0.05) (*n* = 6). Different lowercase letters indicate statistically significant differences among groups (*p* < 0.05).

### 3.5. Water Sorption and Solubility

Table 6 shows the mean and SD of W_sp_ and W_sl_ values for the control and various CNF concentration groups. The W_sp_ of resin increased significantly with CNF addition. Specifically, the control group exhibited the lowest W_sp_ (25.1 µg/mm^3^). Under 2.0 wt% CNF, W_sp_ exhibited the highest value (28.3 µg/mm^3^) but did not differ significantly from the value under 1.5 wt% CNF (*p* = 0.928). W_sl_ remained relatively stable at all CNF concentrations, and no significant differences were identified among the groups based on multiple comparisons (*p* > 0.05).

### 3.6. Regression Analysis of CNF Concentration and Material Properties

**Figure 7:**
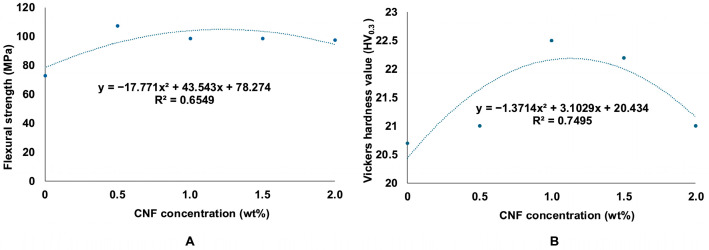
Quadratic regression analyses of (**A**) flexural strength and (**B**) Vickers hardness as a function of CNF concentration. The fitted equations and corresponding coefficients of determination (R^2^) are shown.

To further evaluate the relationship between CNF concentration and mechanical performance, quadratic regression analyses were performed using the mean flexural strength and Vickers hardness values (Figure 7). The fitted models showed coefficients of determination (R^2^) of 0.66 and 0.75 for flexural strength and hardness, respectively. The theoretical maxima predicted by the fitted curves were approximately 1.23 wt% CNF for flexural strength and 1.13 wt% CNF for hardness.

## 4. Discussion

In this in vitro study, we evaluated whether adding CNFs to 3D-printed denture base resin could enhance its mechanical reliability and physical properties. The null hypothesis proposed that CNF incorporation does not significantly affect the properties of the resin composites. While specific parameters, including FM and W_sl_, did not differ significantly across groups, key properties, including FS, VH, ΔE*, and W_sp_, varied significantly across CNF concentrations. These findings led to a partial rejection of the null hypothesis, suggesting that CNF concentrations selectively influence specific physical properties of 3D-printed denture base resins.

The incorporation of CNF significantly enhanced the material’s FS and surface hardness, with optimal reinforcement observed at lower concentrations. The improved fracture resistance is clinically significant, as denture base fractures remain one of the most common complications requiring repair or replacement [10]. Therefore, materials capable of more effectively dispersing functional stresses can extend denture longevity and reduce maintenance requirements, particularly for patients subjected to higher occlusal forces.

The strengthening effect is related to the formation of an interconnected nanofiber web-like structure within the polymer matrix, which facilitates stress transfer and delays crack initiation [34,38]. The high aspect ratio of the nanofibers may also enable microcrack bridging, thereby limiting catastrophic failure. These observations are consistent with those of a previous study on fiber-reinforced polymers, suggesting that these benefits can be achieved even at relatively low filler concentrations [20,26,41,42]. Although the 2.0 wt% group did not exhibit a statistically significant decrease in FS, its strength did not improve further, suggesting that an excess of CNF may lead to agglomeration, thereby limiting stress transfer efficiency.

SEM analysis supported the relationship between nanofiber dispersion and mechanical behavior. The control group exhibited a smooth fracture surface with typical brittle fracture characteristics, whereas the CNF-reinforced groups displayed a rough surface with visible web-like structures, indicating altered stress distribution. These morphological changes indicate altered crack propagation behavior and increased energy dissipation during fracture, which may be related to improved stress transfer between the resin matrix and the nanofibers. Such mechanisms likely contributed to the enhanced flexural properties observed in the CNF-containing groups.

However, the reinforcing effect did not increase proportionally with filler content. Although the mechanical properties improved with the addition of CNF, this improvement tended to plateau or even decline slightly at higher concentrations. SEM observations revealed irregular surface features and pore-like structures in the high-concentration group, indicating that the microstructure was not sufficiently uniform [49]. Although SEM observations alone cannot definitively confirm whether nanofiber agglomeration has occurred, these morphological features may reflect local microstructural inhomogeneities. Such inhomogeneities could act as stress concentration points, thereby adversely affecting mechanical properties [19,21,23]. Similar phenomena have been reported in previous studies on nanofiber-reinforced polymer composites, indicating the importance of achieving uniform dispersion of nanofibers within the polymer matrix [7,50,51].

To promote homogeneous distribution, homogenization and high-speed mixing processes were employed prior to specimen preparation, achieving satisfactory dispersion within the concentration range studied [38,52]. These results indicate that the reinforcing effect of CNF depends not only on the filler content but also on the quality of nanofiber dispersion. Within the evaluated concentration range, 1.0 wt% CNF demonstrated the optimal balance between reinforcing effect and structural integrity. Nevertheless, the use of additional characterization techniques, such as elemental imaging or nanoscale imaging, would help further elucidate the distribution of CNF within the polymer matrix.

The regression analysis shown in Figure 7 further corroborates this observation. The fitted quadratic model predicts that flexural strength and hardness will reach their theoretical maxima at CNF contents of approximately 1.23 wt% and 1.13 wt%, respectively, suggesting that the optimal reinforcement concentration may be close to 1.0 wt% CNF. However, since these estimates are based on a limited number of concentration levels, they should be interpreted with caution.

The FM did not significantly change after CNF incorporation, suggesting that reinforcement improved fracture resistance without increasing material stiffness. The addition of CNF primarily enhances strength-related properties while maintaining the material’s inherent flexibility. This mechanical behavior may be advantageous for denture bases, as overly rigid materials are more prone to sudden fracture [2,13], whereas appropriate flexibility helps to distribute stress more effectively during mastication and cyclic functional loading.

Curry was selected as the staining medium because it is a commonly consumed food and contains strong chromogenic agents, such as turmeric-derived pigments, making it suitable for simulating a clinically relevant staining challenge. Exposure to curry produced a marked color change in the denture base resin, as reflected by increased ΔE* values. Color stability did not follow a linear dose-dependent relationship with CNF concentration. Optimal performance was observed at 1.0 wt%, suggesting that excessive filler content may adversely affect optical homogeneity. Nevertheless, CNF incorporation at optimal concentrations conferred some improvement in color stability, although ΔE values remained relatively elevated under curry staining conditions. The staining effect is largely attributed to curcumin, which penetrates resin microdefects and forms stable bonds, while lipid components may facilitate pigment diffusion. Comparable staining patterns have been described previously, with material composition and reinforcement affecting color stability, consistent with the present results [14,17,53].

The color change observed in this study was partly explained by the water-related behavior of the material. Increased water sorption facilitates pigment penetration into the polymer network, thereby increasing staining susceptibility [18]. CNF incorporation resulted in a concentration-dependent rise in water sorption due to hydrogen bonding between water molecules and the abundant hydroxyl groups of the nanofibers [37]. However, all values remained within the ISO standard [47], indicating that clinical acceptability was preserved.

The influence of CNF concentration on color change is multifactorial. The higher water sorption values observed in the CNF-containing groups were probably related to the hydrophilic behavior of cellulose nanofibers and potential formation of interfacial microvoids, which facilitate water diffusion within the polymer matrix, thereby heightening staining susceptibility. Nanofiber dispersion and interfacial behavior also appear relevant. The amphiphilic surface characteristics of CNFs may have improved compatibility with the resin matrix, promoting more uniform distribution and structural stability. This may explain why CNF concentrations enhanced favorable material performance without markedly compromising color properties [36]. Despite the increase in water sorption, CNF incorporation did not significantly affect water solubility. This suggests that the nanofibers were well integrated within the cross-linked polymer network, limiting the release of soluble components. Strong interfacial bonding likely contributed to the preservation of structural integrity and resistance to mass loss [6,34].

In the present study, all specimens were fabricated using a 0° orientation, aligned parallel to the build platform. This orientation provides superior mechanical properties in DLP-based printing, likely owing to reduced interlayer anisotropy and improved structural integrity. Furthermore, the 0° orientation maximizes dimensional accuracy and surface quality while minimizing the stair-step effect and the need for support structures [54]. Therefore, we adopted a uniform 0° orientation to standardize specimen preparation and isolate the influence of CNF incorporation on material properties. Future research should investigate the interaction between CNF content and print orientation to further optimize mechanical properties and manufacturing efficiency.

This study has some limitations. Despite the anisotropic characteristics associated with additively manufactured materials, we did not investigate the effect of printing orientation in the present study. Although the sample size met the minimum requirements established for the efficacy analysis, the effect size (2.14) was estimated based on pre-test data and may therefore overestimate the true treatment effect. Furthermore, although 1.0 wt% CNF exhibited the most favorable overall performance in this study, the concentration range investigated was relatively broad. Future studies should employ finer concentration increments (e.g., 0.4, 0.5, 0.6, 0.7, 0.8, 0.9, and 1.0 wt%) to more precisely determine the concentration at which mechanical properties reach their optimal values. This study was conducted entirely in vitro and was limited to evaluating the mechanical and physical properties of specimens stored at a constant temperature of 37 °C, corresponding to the intraoral environment. Evaluations involving thermal cycling, fatigue loading, accelerated aging, and biofilm formation were not performed. Therefore, caution should be exercised when extrapolating these findings directly to long-term clinical performance. Future studies should investigate the durability and biological behavior of cellulose nanofiber-reinforced denture base resins under more complex simulated oral conditions, including thermal fluctuations, mechanical fatigue, and aging processes.

## 5. Conclusions

Within the limitations of this in vitro study, the incorporation of cellulose nanofibers (CNFs) affected the mechanical and physical properties of 3D-printed denture base resins. CNF addition improved flexural strength and surface hardness, and 1.0 wt% CNF exhibited the most favorable overall performance among the tested concentrations.

## Figures and Tables

**Table 1:** Materials used.

Material	Product Name	Composition	Manufacturer	Code
3D-printed denture base resin	dima Print Denture Base (Original Pink)	Methacrylic acid-based monomer (40–60 wt%), trimethylolpropane trimethacrylate (3–10 wt%), photopolymerization initiator, stabilizer, pigment	Kulzer GmbH, Hanau, Germany	66081672
CNF powder	BiNFi-s dry powder	Cellulose nanofibers derived from plant pulp	Sugino Machine Co., Ltd., Toyama, Japan	WFo-UNDP

3D, three-dimensional; CNF, cellulose nanofiber.

**Table 2:** The main 3D printing and post-processing parameters.

Parameters	Value	Units
Printing orientation	Horizontal	−
Layer thickness	100	μm
Washing time	10 (99% IPA)	Min
Curing time	20	Min
Curing temperature	60	°C

IPA, Isopropyl Alcohol.

**Table 3:** Flexural strength and modulus (mean ± SD) of the experimental groups at different CNF concentrations (wt%).

CNF Content (wt%)	Flexural Strength (MPa)		*p*-Value of One-Way ANOVA	Flexural Modulus (GPa)		*p*-Value of Welch’s ANOVA
	Mean	SD		Mean	SD	
Control (0)	73.2 ^a^	12.2	<0.001	2.5 ^a^	0.6	0.082
0.5	107.5 ^b^	4.3		3.1 ^a^	0.0	
1.0	98.8 ^b^	5.3		3.1 ^a^	0.0	
1.5	98.7 ^b^	5.6		3.2 ^a^	0.1	
2.0	97.6 ^b^	6.7		3.1 ^a^	0.1	

One-way ANOVA among six groups of flexural strengths yielded *p* < 0.05. ^a,b^ Superscript letters indicate statistically significant differences identified by Tukey’s HSD post hoc test, *p* < 0.05. Welch’s ANOVA among the six groups of flexural moduli revealed no significant differences (*p* = 0.082). HSD, Honestly Significant Difference; CNF, cellulose nanofiber; SD, standard deviation; ANOVA, analysis of variance.

**Table 4:** Mean and SD values of Vickers hardness value (HV_0.3_) for the test groups with different CNF concentrations (wt%).

CNF Content (wt%)	HV_0.3_		*p*-Value of One-Way ANOVA
	Mean (HV_0.3_)	SD	
Control (0)	20.7 ^a^	0.7	<0.001
0.5	21.0 ^a^	0.8	
1.0	22.5 ^b^	0.8	
1.5	22.2 ^b^	0.8	
2.0	21.0 ^a^	1.0	

Variations in Vickers hardness (HV_0.3_) were detected among the six groups using one-way analysis of variance. ^a,b^ Different superscript letters denote significant differences based on Tukey’s HSD multiple comparison procedure (*p* < 0.05). HSD, Honestly Significant Difference; CNF, cellulose nanofiber; SD, standard deviation.

**Table 6:** Mean and SD values of water sorption (W_sp_) and water solubility (W_sl_) for the test groups with different CNF concentrations (wt%).

CNF Content (wt%)	W_sp_		*p*-Value of One-Way ANOVA	W_sl_		*p*-Value of One-Way ANOVA
	Mean (µg/mm^3^)	SD		Mean (µg/mm^3^)	SD	
Control (0)	25.1 ^a^	0.5	<0.001	1.0 ^a^	0.4	0.821
0.5	26.0 ^b^	0.7		1.1 ^a^	0.3	
1.0	26.7 ^bc^	0.5		1.1 ^a^	0.3	
1.5	27.1 ^cd^	0.3		1.0 ^a^	0.3	
2.0	28.3 ^d^	0.3		0.9 ^a^	0.4	

One-way ANOVA among the six groups of W_sp_ and W_sl_ yielded *p* < 0.05. ^a–d^ Superscript letters indicate significant differences based on Tukey’s HSD test for post hoc comparison tests at *p* < 0.05. CNF, cellulose nanofiber; HSD, Honestly Significant Difference; ANOVA, analysis of variance; SD, standard deviation.

## Data Availability

The original contributions presented in this study are included in the article. Further inquiries can be directed to the corresponding author.

## Abbreviations

The following abbreviations are used in this manuscript:

3D	three-dimensional
DLP	digital light processing
CNF	cellulose nanofiber
IPA	isopropyl alcohol
HV	Vickers hardness
